# FGM-based remote intervention for adults with type 1 diabetes: The FRIEND randomized clinical trial

**DOI:** 10.3389/fendo.2022.1054697

**Published:** 2022-11-25

**Authors:** Jinju Lee, Myeong Hoon Lee, Jiyun Park, Kyung-Soo Kim, Soo-Kyung Kim, Yong-Wook Cho, Hyun Wook Han, Young Shin Song

**Affiliations:** ^1^ Department of Biomedical Science, Graduate School, CHA University, Seongnam, South Korea; ^2^ Institute for Biomedical Informatics, CHA University School of Medicine, CHA University, Seongnam, South Korea; ^3^ Department of Internal Medicine, CHA Bundang Medical Center, CHA University School of Medicine, Seongnam, South Korea

**Keywords:** remote consultation, telemedicine, blood glucose self-monitoring, insulin, diabetes mellitus, randomized controlled trial

## Abstract

**Background:**

The use of flash glucose monitoring (FGM) in conjunction with proper education has been reported to improve glycemic control in people with diabetes on insulin therapy. However, there are still few randomized controlled trials on the educational effect, and an ideal educational model has not been established. This study aimed to estimate the efficacy of remote intervention for glycemic control in adults with type 1 diabetes using FGM.

**Methods:**

In this single-center, randomized controlled trial, we enrolled adults with type 1 diabetes (HbA1c ≥7.0%). The participants were randomly assigned (1:1) to either FGM use with remote intervention (intervention group) or FGM use only (control group). Changes in glycemic outcomes such as HbA1c levels and continuous glucose monitoring metrics were evaluated at 12 weeks.

**Results:**

Among 36 randomized participants (mean age, 44.3 years; mean baseline HbA1c, 8.9%), 34 completed the study. The remote intervention did not significantly reduce HbA1c levels. FGM use significantly improved HbA1c levels by −1.4% and −0.8% in both groups with and without remote intervention, respectively (*P*=0.003 and *P*=0.004, respectively). However, the intervention group showed significant increases in time with glucose in the range of 70–180 mg/dL (TIR; from 49.8% to 60.9%, *P*=0.001) and significant decreases in time with hyperglycemia (*P*=0.002) and mean glucose (*P*=0.017), but the control group did not. Moreover, the TIR (*P*=0.019), time with hyperglycemia >250 mg/dL (*P*=0.019), and coefficient of variation (*P*=0.018) were significantly improved in the intervention group compared to the control group. In particular, the CGM metrics improved gradually as the remote intervention was repeated. Furthermore, the intervention group reported higher treatment satisfaction (*P*=0.016).

**Conclusions:**

Ongoing, personalized education during FGM use may lead to amelioration of glycemic control in adults with type 1 diabetes, even remotely.

**Clinical trial registration:**

https://clinicaltrials.gov/ct2/show/NCT04936633, identifier NCT04936633.

## Introduction

Multiple randomized controlled trials (RCTs) assessing real-time continuous glucose monitoring (rtCGM) or flash glucose monitoring (FGM) have demonstrated an effect on reducing glycated hemoglobin (HbA1c) levels and/or rates of hypoglycemia in patients with diabetes using intensive insulin regimens (multiple daily injections [MDI] or continuous subcutaneous insulin infusion [CSII]) (1–3). Based on accumulating evidence, the clinical practice guidelines for diabetes (4–6) now recommend using rtCGM or FGM for diabetic management in these patients. Previous studies, however, have shown that the use of rtCGM or FGM without adequate education has led to only modest or partial improvement of outcomes. In a meta-analysis of RCTs comparing rtCGM to usual methods of care in type 1 and type 2 diabetes (7), the use of rtCGM and FGM resulted in a modest (0.23%) or no reduction in HbA1c, respectively, and the use of FGM also resulted in no reduction in time with hyperglycemia.

FGM, also known as intermittently scanned CGM, continuously measures interstitial glucose levels but requires scanning to store the obtained glucose values. Most RCTs evaluating the efficacy of FGM did not indicate an outcome of HbA1c reduction (3, 8–10) except for one RCT involving type 2 diabetes (11). The design of this trial differed from others in that patients were educated about insulin dose adjustment and carbohydrate counting during the study period. Furthermore, a previous RCT on FGM users with type 1 and insulin-treated type 2 diabetes identified the effectiveness of a structured education program, termed FLASH, by comparing educated and uneducated groups (12). Taken together, findings of the previous studies suggested the importance of adequate education for patients using FGM.

Evidence indicating the importance of education is still lacking and an ideal educational model has not been established. The aim of this study was to determine the efficacy of remote intervention for glycemic control in adults with type 1 diabetes using FGM.

## Materials and methods

### Study design and participants

The FGM data-based Remote IntervEntion for adults with insuliN-dependent Diabetes (FRIEND) trial was a 12-week, investigator-initiated, open-label, parallel, randomized controlled study conducted at the CHA Bundang Medical Center in Korea. The study protocol (**Supplementary Figure 1**) was approved by the Institutional Review Board of CHA Bundang Medical Center (no. 2021-03-032) and performed in accordance with the Declaration of Helsinki and the Good Clinical Practice Guidelines of the International Council for Harmonization. All the participants provided written informed consent before any trial-related activity. The study was registered at ClinicalTrial.gov (trial number, NCT04936633).

Eligible subjects were adults with type 1 diabetes aged 19–75 years who had been on intensive treatment with MDI or CSII therapy for more than one year, had a HbA1c level of 7.0% or higher, and a desire to use the FGM system. The exclusion criteria were non-insulin-dependent diabetes, diabetes duration <1 year, a history of using a rtCGM or FGM within the previous 12 weeks, pregnancy, end-stage renal disease and on dialysis, current treatment for severe cognitive impairment or psychiatric problems, a history of substance abuse or alcoholism within the previous 12 weeks, a history of corticosteroid therapy for more than seven consecutive days within the previous four weeks, and participation in other clinical trials within the previous four weeks. The flowchart of study participants is shown in **Supplementary Figure 2**.

### Randomization and procedures

The participants were randomly assigned in a 1:1 ratio to either the intervention group or the control group. The intervention group used FGM for 12 weeks with remote intervention by medical staff and the control group used FGM without intervention. The participants were stratified at randomization according to their baseline HbA1c level (<9.0% or ≥9.0%) and age (<47 or ≥47 years).

All participants were provided with a FGM system (FreeStyle Libre; Abbott Diabetes Care, Witney, UK) with basic instructions on how to use it. In the intervention group, the remote intervention was conducted over a phone call at 2-week intervals for a total of five times during the study period if one or more of the following criteria were met in the previous two weeks; i.e., active time of sensor <70%, the number of scans per day <4, time with glucose in the range of 70–180 mg/dL <70%, time with glucose below 70 mg/dL ≥4%, time with glucose below 54 mg/dL ≥1%, time with glucose above 180 mg/dL ≥25%, time with glucose above 250 mg/dL ≥5%, coefficient of variation ≥33%, and mean glucose level ≥140 mg/dL.

The remote intervention lasted about 10 minutes and was based on CGM data in LibreView. Its contents were as follows: education on the insulin to carbohydrate ratio and the insulin sensitivity factor; carbohydrate counting training; insulin management training including basal dose adjustment, bolus dose titration based on meal content and current glucose level, and use of a sliding scale; identifying the causes of hypoglycemia, hyperglycemia, or glycemic variability; advice on lifestyle modifications such as diet and exercise; and how to use FGM system including using glucose trend arrows. When the sensor activation time was less than 70% during the previous two weeks, both groups received phone calls or text messages encouraging FGM use.

At baseline and week 12, blood samples were taken from all participants to determine HbA1c levels and questionnaires were completed on following characteristics: treatment satisfaction and perception of hyperglycemia or hypoglycemia (Diabetes Treatment Satisfaction Questionnaire [status version and change version, DTSQs and DTSQc], Korean ver. 8.3.06, licence ref CB1202) (13), depression (Patient Health Questionnaire-9, PHQ-9) (14), and anxiety (General Anxiety Disorder-7, GAD-7) (15). The DTSQ questionnaire consists of eight questions with six querying treatment satisfaction, one querying perceived frequency of hyperglycemia, and one querying perceived frequency of hypoglycemia. Higher scores on six items asking about treatment satisfaction indicate greater satisfaction with treatment. Lower scores on two items asking about the perceived frequency of hyperglycemia and hypoglycemia indicate that blood glucose levels were closer to the ideal, while higher scores indicate problems. The PHQ-9 and GAD-7 questionnaires consist of 9 and 7 questions, respectively, with higher scores indicating severe depression or anxiety.

### Outcomes

The primary outcome of the study was changes in HbA1c levels from baseline to week 12. Secondary glycemic outcomes included changes in CGM metrics, such as time with glucose in a range of 70–180 mg/dL (TIR), time with hypoglycemia (<54 and <70 mg/dL), time with hyperglycemia (>180 and >250 mg/dL), mean glucose level, and coefficient of variation (CV). CV (%) was calculated using the following formula: dividing the standard deviation (SD) of glucose levels by the mean glucose level and multiplying by 100. The CGM metrics data of the first two weeks and two weeks before week 12 were compared. In addition, changes in the psychosocial, behavioral, and physical variables were assessed as outcomes; i.e., scores of the DTSQs, DTSQc, PHQ-9, and GAD-7 questionnaires, total daily doses of insulin, the number of scans per day, lifestyle factors such as diet and exercise, and anthropometric variables.

### Statistical analysis

Among the baseline characteristics of study participants, comparisons of categorical variables were performed with either Pearson’s chi-squared test or Fisher’s exact test, as appropriate. For continuous variables, either the independent *t*-test or the Wilcoxon-Mann-Whitney test was used, as appropriate. The Shapiro-Wilk test and skewness/kurtosis test were used to test for the normality of data. The outcomes comparing baseline and 12-week follow-up data for each group were analyzed with the paired *t*-test or the Wilcoxon signed-rank test. The analysis of covariance (ANCOVA) was used to compare changes in continuous variables between groups after adjusting for the baseline values, and the rank transform ANCOVA was used when data violated the ANCOVA assumptions. To test the relationship between two variables, either Pearson’s or Spearman’s correlation analysis was used. For the CGM metrics, *post hoc* analysis was performed separately for daytime (6:00 AM–11:59 PM) and nighttime (12:00 AM–5:59 AM) as well as for 2-week durations at baseline and at weeks 4, 8, and 12. The trend of changes in the CGM metrics with an increasing number of study weeks was evaluated using the one-way analysis of variance and test for linearity. Data are presented as number (%), mean ± SD, or median (interquartile range [IQR]). Statistical significance was defined as 2-sided *P* values <0.05. Statistical analyses were performed using R Statistical Software (v4.1.1; R Core Team 2021).

## Results

### Baseline characteristics

Participants were recruited between June 2021 and December 2021. A total of 36 participants were randomly assigned to the intervention group (n = 18) or the control group (n = 18; **Supplementary Figure 2**). A total of 34 participants (94%) with 17 in each group completed the study and were analyzed for the per-protocol population. All participants in the intervention group had five times of remote intervention because they met ≥1 intervention criteria every two weeks.

The baseline characteristics of participants are shown in **Table 1**. There were no significant differences in baseline demographics and clinical characteristics between the two groups. The mean age of participants was 44.3 (SD, 13.3) years and 52.8% were female. The mean duration of diabetes was 17.1 (SD, 10.4) years and the mean baseline HbA1c level was 8.9% (SD, 1.6).

**Table 1:** Baseline characteristics of study participants.

Characteristics	Intervention (n = 18)	Control (n = 18)	*P*
Age, years	45.4 ± 12.3	43.1 ± 14.6	0.607
Sex			0.504
Female	8 (44.4)	11 (61.1)	
Male	10 (55.6)	7 (38.9)	
Body mass index, kg/m^2^	23.9 (20.9–26.4)	22.6 (20.1–25.4)	0.481
Waist circumference, cm	84.2 ± 11.2	82.9 ± 13.0	0.749
Duration of diabetes, years	16.0 ± 10.4	18.2 ± 10.5	0.543
HbA1c, %	9.2 ± 2.0	8.6 ± 1.1	0.251
Fasting blood glucose, mg/dL	124.0 (90.2−145.8)	141.5 (97.5−201.0)	0.343
C-peptide, ng/mL	0.3 ± 0.4	0.2 ± 0.4	0.436
Type of insulin therapy			0.486
Multiple daily insulin injections	18 (100.0)	16 (88.9)	
Continuous subcutaneous insulin infusion	0 (0.0)	2 (11.1)	
Duration of insulin use, years	12.6 (4.8–19.4)	13.7 (7.1–23.0)	0.406
Total daily dose of insulin, units	40.5 (28.0–62.5)	42.5 (38.5–61.8)	0.601
≥1 Diabetes-related complications	8 (44.4)	10 (55.6)	0.739
≥1 Diabetic education history	15 (83.3)	15 (83.3)	1.000
Highest education			0.587
Less than middle school	2 (11.1)	1 (5.6)	
High school	4 (22.2)	7 (38.9)	
More than bachelor**’**s degree	12 (66.7)	10 (55.6)	
Smoking status			0.862
Current	9 (50.0)	7 (38.9)	
Ex-smoker	1 (5.6)	1 (5.6)	
Never	8 (44.4)	9 (50.0)	

Values are presented as the mean ± standard deviation, median (interquartile range), or number (%). HbA1c, glycated hemoglobin.

### Changes in HbA1c levels

**Figure 1:**
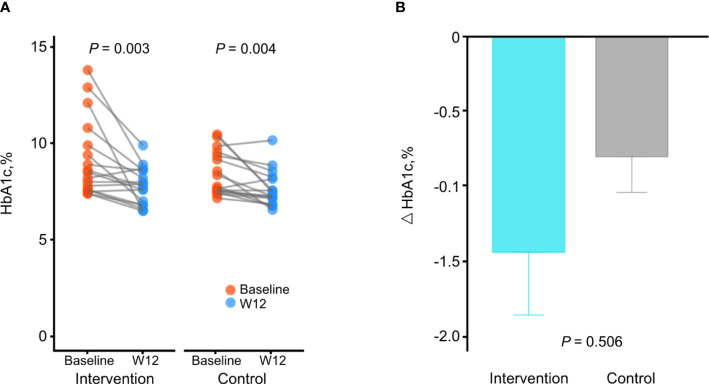
Changes in HbA1c levels during the study. **(A)** Comparison of HbA1c levels between baseline and week 12 in each group. *P* by paired *t*-test. **(B)** Comparison of changes of HbA1c levels between the intervention group and the control group. *P* adjusted for baseline values using analysis of covariance. Data are presented as the mean ± SE.

The changes in HbA1c levels from baseline to week 12 were significant in both groups (from 9.2% ± 2.0% to 7.8% ± 1.0%, *P* = 0.003 in the intervention group; from 8.6% ± 1.1% to 7.8% ± 0.9%, *P* = 0.004 in the control group; **Table 2** and **Figure 1A**). The mean reduction in HbA1c levels was greater in the intervention group compared to the control group (−1.4% and −0.8%, respectively), although the difference between groups were not statistically significant (*P* adjusted for baseline values = 0.506; **Figure 1B**).

**Table 2:** Changes in HbA1c levels and continuous glucose monitoring metrics.

	Baseline	Week 12	Change from baseline (95% CI)	*P*	Adjusted difference between groups (95% CI)	*P*
HbA1c, %						
Intervention	9.2 ± 2.0	7.8 ± 1.0	−1.4 (−2.3 to −0.5)	**0.003**	−0.2 (−0.8 to 0.4)	0.506
Control	8.6 ± 1.1	7.8 ± 0.9	−0.8 (−1.3 to −0.3)	**0.004**		
**Continuous glucose monitoring outcomes**						
Time with glucose 70–180 mg/dL, %						
Intervention	49.8 ± 15.7	60.9 ± 7.9	11.1 (5.1 to 17.1)	**0.001**	7.0 (1.2 to 12.7)	**0.019**
Control	50.0 ± 15.7	54.0 ± 13.9	4.0 (−1.6 to 9.6)	0.151		
Time with glucose <54 mg/dL, %						
Intervention	0.4 (0.0–0.7)	0.1 (0.0–1.0)	−0.1 (−1.4 to 1.4)	0.708	−0.5 (−6.0 to 5.1)	0.863
Control	0.1 (0.0–2.5)	0.5 (0.0–1.3)	−0.7 (−4.7 to 0.4)	0.726		
Time with glucose <70 mg/dL, %						
Intervention	4.1 (0.6–7.5)	3.1 (1.0–7.6)	0.2 (−1.9 to 2.4)	0.973	−2.1 (−7.2 to 2.9)	0.393
Control	3.4 (1.9–6.5)	4.9 (1.7–10.3)	1.3 (−4.7 to 5.2)	0.491		
Time with glucose >180 mg/dL, %						
Intervention	44.6 ± 19.2	33.3 ± 11.3	−11.4 (−18.1 to −4.7)	**0.002**	−6.3 (−14.2 to 1.5)	0.110
Control	43.2 ± 18.7	38.8 ± 17.8	−4.4 (−12.2 to 3.5)	0.256		
Time with glucose >250 mg/dL, %						
Intervention	18.8 ± 17.3	9.9 ± 5.1	−8.9 (−16.6 to −1.2)	**0.026**	−7.2 (−13.2 to −1.3)	**0.019**
Control	19.0 ± 15.1	17.2 ± 14.2	−1.8 (−6.9 to 3.4)	0.480		
Mean glucose, mg/dL						
Intervention	180.1 ± 43.3	159.4 ± 21.9	−20.7 (−37.1 to −4.2)	**0.017**	−11.9 (−29.2 to 5.4)	0.170
Control	178.6 ± 42.1	170.6 ± 40.1	−8.0 (−24.9 to 8.9)	0.331		
Coefficient of variation, %						
Intervention	40.8 ± 6.5	39.0 ± 7.8	−1.8 (−4.3 to 0.7)	0.151	−6.4 (−11.5 to −1.2)	**0.018**
Control	42.7 ± 9.3	43.1 ± 6.3	0.4 (−2.6 to 3.4)	0.791		

Total of 34 participants (17 in each group) who completed the 12-week study were analyzed. Values of baseline and week 12 are presented as the mean ± standard deviation or median (interquartile range). The change at week 12 from baseline in each group was evaluated using paired *t*-test or Wilcoxon signed-rank test for parametric or non-parametric data, and was presented as the mean or median, respectively. The baseline corrected difference between groups was evaluated using analysis of covariance (ANCOVA) or rank transform ANCOVA, depending on whether the ANCOVA assumptions were met, and was presented as the mean of values or mean residual of rank-transformed values, respectively. Significant *P* values in bold. CI, confidence interval; HbA1c, glycated hemoglobin.

Although changes in HbA1c levels were not significantly different between groups and both groups showed significant changes in HbA1c levels, only the intervention group showed significant correlations between changes in HbA1c levels and changes in CGM metrics, such as TIR (R = 0.640, *P* = 0.006), time with glucose >180 mg/dL (R = −0.710, *P* = 0.001), and mean glucose level (R = −0.670, *P* = 0.005), whereas the control group did not show a correlation (**Supplementary Figure 3**). This result suggests that HbA1c levels were improved along with the CGM metrics in the intervention group.

### Changes in continuous glucose monitoring metrics

**Figure 2:**
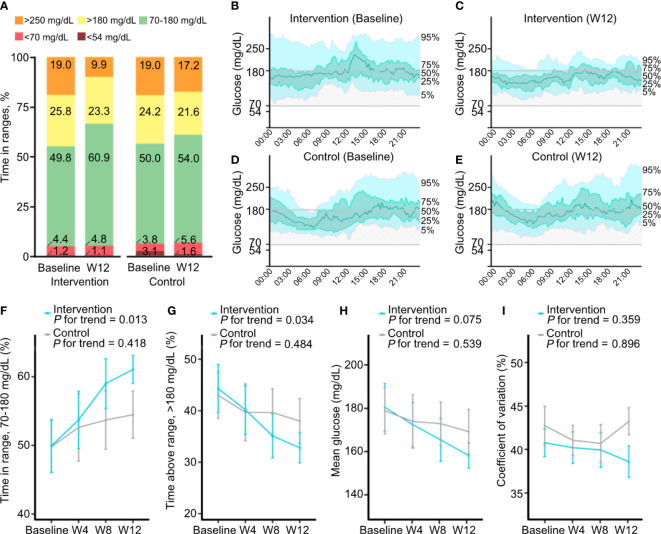
Changes in continuous glucose monitoring metrics during the study. **(A)** Mean percentages of time with glucose in ranges of <54, <70, 70–180, >180, and >250 mg/dL at baseline and week 12 in the intervention and control groups. **(B–E)** Ambulatory glucose profiles, which are summaries of glucose values from 14 consecutive days at baseline and at week 12, in the intervention group **(B, C)** and the control group **(D, E)**. The median line (green line) is surrounded by the interquartile range (25th to 75th percentile, shaded in light green) and the interdecile range (10th to 90th percentile, shaded in light blue). **(F–I)** Changes in continuous glucose monitoring metrics at 4-week intervals. Mean percentages of time with glucose in ranges of 70–180 **(F)** and >180 mg/dL **(G)**, mean glucose **(H)**, and coefficient of variation **(I)** at baseline and at weeks 4, 8, and 12 in the intervention and control groups. Data are presented as the mean ± SE.

The TIR significantly increased from baseline to week 12 in the intervention group (from 49.8% ± 15.7% to 60.9% ± 7.9%; *P* = 0.001), but not in the control group (from 50.0% ± 15.7% to 54.0% ± 13.9%, *P* = 0.151; **Table 2** and **Figure 2A**). Participants in the intervention group showed significant decreases in time with hyperglycemia (*P* = 0.002 and *P* = 0.026 for >180 and >250 mg/dL, respectively) and mean glucose level (*P* = 0.017), whereas those in the control group did not show significant changes. The changes in time with hypoglycemia (<54 and <70 mg/dL) and glycemic variability measured by CV from baseline were not significant in either group. When we compared the CGM metrics between two groups, changes in the TIR (adjusted mean difference, 7.0%, *P* = 0.019), time with glucose >250 mg/dL (adjusted mean difference, −7.2 mg/dL, *P* = 0.019), and CV (adjusted mean difference, −6.4%, *P* = 0.018) were significantly improved in the intervention group compared to the control group (**Table 2**). Moreover, the ambulatory glucose profile (AGP) at week 12 compared to baseline showed that the IQR and the interdecile range (5th to 95th percentile) of glucose levels were narrower and the median was stabilized in the intervention group, indicating a reduction in glycemic variability (**Figures 2B–E**).

### *Post hoc* analyses of continuous glucose monitoring metrics

The CGM metrics at daytime and nighttime and at 4-week intervals were obtained. Overall, the changes in glycemic metrics during both daytime and nighttime were similar to those of all day (**Supplementary Table 1**). During the daytime, significant decreases in time with glucose >180 mg/dL and mean glucose level were found in the intervention group. During the nighttime, a significant increase in TIR and a significant decrease in time with glucose >250 mg/dL were observed in the intervention group. Although statistical significance was lacking due to a low percentage of time with hypoglycemia, time with nocturnal hypoglycemia (<70 mg/dL at nighttime) was decreased in the intervention group, but it was increased in the control group.

The changes in glycemic variability measured by CV from baseline were decreased in the intervention group, contrary to the increase in the control group, but the changes were not significant in either group. However, the between-group difference of the change in CV was significantly reduced in the intervention group compared to the control group both during the daytime and nighttime (adjusted mean difference, −5.6% and −7.0%, *P* = 0.039 and *P* = 0.022, respectively; **Supplementary Table 1**).

Furthermore, in the analysis of glycemic metrics at 4-week intervals, unlike the control group, there was an increasing trend of TIR (*P* for trend = 0.013) and a decreasing trend of time with hyperglycemia >180 mg/dL (*P* for trend = 0.034) as the length of time participants in the intervention group used FGM with remote intervention increasing (**Figures 2F, G**; **Supplementary Table 2**). Mean glucose levels and CV decreased gradually in the intervention group, although the linear trend was not statistically significant (**Figures 2H, I**).

### Changes in psychosocial, behavioral, and physical variables

Regarding DTSQs questionnaire items, the satisfaction-related scores increased from baseline at week 12 in both groups, though not significantly (**Table 3**). The perceived hyperglycemia-related scores decreased significantly in the intervention group (*P* = 0.015) and the perceived hypoglycemia-related scores increased significantly in the control group (*P* = 0.041), although the between-group differences were not significant. However, the DTSQc results at week 12 showed that the satisfaction-related scores were significantly higher in the intervention group than the control group (*P* = 0.016). Although the decrease in PHQ-9 scores was significant in both groups and the decrease in GAD-7 scores was not, the intervention group showed a greater decrease than the control group, indicating that anxiety and depression were further reduced in the intervention group.

**Table 3:** Changes in psychosocial outcomes.

	Baseline	Week 12	Change from baseline (95% CI)	*P*	Adjusted difference between groups (95% CI)	*P*
**Psychosocial outcomes**						
DTSQs score						
Treatment satisfaction						
Intervention	26.6 ± 7.8	28.2 ± 6.7	1.6 (−1.2 to 4.4)	0.243	0.2 (−3.2 to 3.5)	0.917
Control	25.4 ± 5.0	27.3 ± 5.2	1.9 (−0.9 to 4.8)	0.163		
Perceived hyperglycemia						
Intervention	4.2 ± 1.6	3.1 ± 1.8	−1.1 (−1.9 to −0.2)	**0.015**	−1.3 (−2.5 to 0.0)	0.056
Control	2.8 ± 1.0	3.7 ± 1.6	0.9 (0.0 to 1.9)	0.060		
Perceived hypoglycemia						
Intervention	2.5 ± 1.5	2.6 ± 1.5	0.1 (−0.6 to 0.9)	0.743	−0.8 (−1.9 to 0.3)	0.139
Control	2.1 ± 1.5	3.3 ± 1.7	1.2 (0.1 to 2.3)	**0.041**		
DTSQc score^a^						
Treatment satisfaction						
Intervention		16.1 ± 2.3			2.5 (0.5 to 4.5)^b^	**0.016** ^b^
Control		13.6 ± 3.3				
Perceived hyperglycemia						
Intervention		0.9 ± 2.2			0.1 (−1.2 to 1.5)^b^	0.789^b^
Control		0.8 ± 1.5				
Perceived hypoglycemia						
Intervention		0.1 ± 1.8			−1.0 (−2.1 to 0.1)^b^	0.063^b^
Control		1.1 ± 1.1				
Depression, PHQ-9 score						
Intervention	6.8 ± 4.5	3.9 ± 3.5	−2.9 (−5.1 to −0.6)	**0.015**	−1.3 (−3.4 to 0.8)	0.214
Control	8.5 ± 6.1	6.3 ± 5.3	−2.2 (−3.4 to −1.0)	**0.002**		
Anxiety, GAD-7 score						
Intervention	3.9 ± 3.8	2.5 ± 3.4	−1.4 (−2.9 to 0.2)	0.081	−1.0 (−3.2 to 1.2)	0.362
Control	4.9 ± 5.1	4.3 ± 5.5	−0.6 (−2.4 to 1.2)	0.496		

Values of baseline and week 12 are presented as the mean ± standard deviation. Values of the change at week 12 from baseline and the adjusted difference between groups are presented as the mean. The change from baseline in each group was evaluated using paired *t*-test. The baseline corrected difference between groups was evaluated using analysis of covariance. Significant *P* values in bold. DTSQs, The Diabetes Treatment Satisfaction Questionnaire status version. DTSQc, The Diabetes Treatment Satisfaction Questionnaire change version; PHQ-9, Patient Health Questionnaire-9; GAD-7, General Anxiety Disorder-7. ^a^Data for the DTSQc was collected only at week 12. ^b^For the DTSQc score, the unadjusted mean difference between groups were presented. The 95% CI and *P* value were calculated with an independent *t*-test.

The increase in total daily insulin doses was greater in the intervention group, even though there was no statistical significance (**Table 4**). The number of scans per day was reduced significantly in the control group (*P* = 0.034), but not in the intervention group. The frequency of meals, snacks, and exercise were not changed in either group, and the hours of exercise decreased significantly more in the control group (*P* = 0.042). Body mass index and waist circumference increased in both groups, and only the increase in the body mass index in the control group was statistically significant (*P* = 0.015).

**Table 4:** Changes in behavioral and physical outcomes.

	Baseline	Week 12	Change from baseline (95% CI)	*P*	Adjusted difference between groups (95% CI)	*P*
**Behavioral outcomes**						
Total daily insulin dose, U						
Intervention	47.6 ± 23.7	50.1 ± 15.0	3.1 (−0.4 to 6.5)	0.079	2.1 (−1.6 to 5.8)	0.256
Control	49.0 ± 14.1	50.1 ± 15.0	1.1 (−0.8 to 2.9)	0.245		
Number of scans per day						
Intervention	11.0 ± 7.3	8.4 ± 4.1	−2.7 (−5.9 to 0.6)	0.103	0.1 (−2.0 to 2.2)	0.712
Control	9.7 ± 3.6	7.4 ± 2.5	−2.3 (−4.4 to −0.2)	**0.034**		
Number of meals per day						
Intervention	2.6 ± 0.5	2.6 ± 0.5	0.0 (0.0 to 0.0)	–	0.0 (0.0 to 0.0)	–
Control	2.5 ± 0.5	2.5 ± 0.5	0.0 (−0.2 to 0.2)	1.000		
Number of snacks per day						
Intervention	0.9 ± 1.0	1.0 ± 1.0	0.1 (−0.1 to 0.2)	0.332	0.1 (−0.1 to 0.3)	0.151
Control	0.6 ± 0.8	0.6 ± 0.8	−0.1 (−0.2 to 0.1)	0.332		
Number of exercise per week						
Intervention	3.9 ± 2.5	2.8 ± 2.4	−1.0 (−3.0 to 0.0)	0.098	1.0 (−0.4 to 2.5)	0.100
Control	2.5 ± 2.7	1.2 ± 2.1	0.0 (−3.0 to 0.0)	0.052		
Hours of exercise per week						
Intervention	3.0 (0.6–5.0)	2.0 (0.0–3.0)	−4.2 (−8.5 to 2.5)	0.139	6.6 (0.3 to 12.9)	**0.042**
Control	1.5 (0.0–3.5)	0.0 (0.0–1.5)	−3.5 (−6.5 to 0.0)	**0.042**		
**Physical outcomes**						
Body mass index, kg/m^2^						
Intervention	23.5 (20.8–26.7)	24.6 (20.9–27.3)	0.5 (0.0 to 1.2)	0.057	0.6 (−1.0 to 2.3)	0.445
Control	23.1 (21.0–25.7)	23.8 (21.2–25.3)	0.6 (0.1 to 1.0)	**0.015**		
Waist circumference, cm						
Intervention	83.9 ± 11.5	84.5 ± 13.0	0.6 (−1.1 to 2.4)	0.445	−0.9 (−3.2 to 1.3)	0.399
Control	83.8 ± 12.9	85.4 ± 13.7	1.6 (0.0 to 3.2)	0.052		

Values of baseline and week 12 are presented as the mean ± standard deviation or median (interquartile range). The change at week 12 from baseline in each group was evaluated using paired *t*-test or Wilcoxon signed-rank test for parametric or non-parametric data, and was presented as the mean or median, respectively. The baseline corrected difference between groups was evaluated using analysis of covariance (ANCOVA) or rank transform ANCOVA, depending on whether the ANCOVA assumptions were met, and was presented as the mean of values or mean residual of rank-transformed values, respectively. Significant *P* values in bold.

## Discussion

In this 12-week RCT, the remote intervention for adults with type 1 diabetes using FGM did not significantly reduce HbA1c levels. The FGM use significantly improved HbA1c levels by −1.4% and −0.8% in the two groups with and without remote intervention, respectively. However, the TIR, time with hyperglycemia >250 mg/dL, and CV were significantly improved by the remote intervention. In particular, as the remote intervention performed repeatedly, there was a significant trend toward the progressive improvement of CGM metrics such as the TIR and time with hyperglycemia >180 mg/dL. Furthermore, the intervention group reported significantly higher levels of treatment satisfaction compared to the control group.

A previous RCT consisting of 216 patients with diabetes on intensive insulin therapy found that the FLASH education program with FGM use improved glycemic control (12). The FLASH curriculum was a group based, 6-week educational program that consisted of four 90-minute sessions. The FLASH program resulted in a 0.3% reduction in HbA1c levels with a 1.8% (26 min/day) increase in TIR at the 6-month follow-up. Another recent RCT that included 47 poorly controlled patients with type 1 diabetes using rtCGM showed improvement of glycemic outcomes with the structured education (16). Structured individualized education was delivered during a 12-week study period in three sessions with two in person and one by phone call, each lasting 30 to 120 minutes. The educated group had a 1.2% reduction in HbA1c levels with a 2.3% (33 min/day) increase in TIR at the 12-week follow-up. Our study showed that remote intervention produced a 1.4% reduction in HbA1c levels with a 11.1% (2 h 40 min/day) increase in TIR from baseline at 12 weeks. Therefore, the remote intervention of our study can be considered to be an effective educational model. Taken together, our results further reinforced the importance of education, and one-on-one education could be more effective than group education for insulin-treated patients using CGM. The findings of these studies on educational effects are sources of evidence and should be detailed in future guidelines.

The control group of our study that only using FGM without remote intervention showed significant improvement in HbA1c levels, which was different from the absence or modest effect of FGM in previous RCTs (3, 8, 9, 11, 12, 16). As possible explanations for this considerable efficacy of FGM, we enrolled participants with poorly controlled diabetes (HbA1c ≥7.0%) They may have neglected self-management, including self-monitoring of blood glucose, before the study. However, in contrast to the intervention group, this HbA1c level reduction in the control group was not associated with changes in CGM metrics, and no variables improved among the CGM metrics. Moreover, although there was no difference between groups in HbA1c improvements, the CGM metrics such as TIR and CV were significantly improved in the intervention group compared to the control group. This may be due to remote intervention lowering the rate of hypoglycemia as well as hyperglycemia. Therefore, although FGM may help lower HbA1c levels in poorly controlled patients with type 1 diabetes, patient education and monitoring are essential to achieve the original goal of CGM, such as reducing glycemic variability.

A major factor contributing to glycemic improvement in the intervention group in our study might be the education for insulin dose adjustment. Although statistical significance was lacking, the total daily dose of insulin increased more in the intervention group than in the control group. Moreover, especially the time with hyperglycemia and the AGP interdecile range were reduced considerably, indicating the effect of individualized education on the appropriate dose of prandial insulin, which prevented wide glycemic excursions. On the other hand, no improvements in diet, exercise, body mass index, and waist circumference were found.

Recently, digital health has played an increasingly important role in diabetes care. A meta-analysis of 32 RCTs evaluating the effectiveness of telemedicine interventions for gestational diabetes demonstrated reduction of not only glycemic levels of patients but also maternal and neonatal/fetal complications (17). In this regard, remote intervention based on CGM data is expected to be effective and will be a promising educational method for CGM users.

To the best of our knowledge, this is the first RCT that assessed the effectiveness of one-on-one education, especially remote intervention, in adults with type 1 diabetes using FGM. The previous RCTs comparing FGM and rtCGM revealed that FGM had less favorable glycemic control outcomes (18–21). Nevertheless, we demonstrated the benefits of individualized remote intervention for FGM users. One of the strengths of this study is the fact that the *post hoc* analysis was performed considering both daytime and nighttime, as well as a monthly time series. In particular, the CGM metrics improved gradually as the remote intervention was repeated, showing the importance of continuous patient monitoring and education based on the patient’s retrospective CGM data. Another strength is that, in addition to glycemic outcomes, various variables such as psychosocial, behavioral, and physical outcomes were investigated.

This study has some limitations. First, the number of participants was small; thus, the statistical power of differences between groups may have been undermined. Nevertheless, it was sufficient to test the outcomes of changes at week 12 from baseline in each group, calculating power based on our HbA1c results would require 28 subjects (14 in each group) at the desired 80% power and an alpha level of 0.05 (2-tailed). Thus, we showed the change from baseline at week 12 as well as the baseline-adjusted difference between groups. Second, although we used stratified randomization to assign the same number of participants to each group based on baseline HbA1c levels (<9.0% or ≥9.0%), the intervention group had a slightly higher mean baseline HbA1c level with a larger SD than the control group. The difference in baseline HbA1c levels between the groups, however, was not statistically significant, and both groups showed significant improvement in HbA1c levels at week 12. Third, as a single-center study, it may not be representative of the Korean general population. Moreover, there may be a bias because the structured education was provided by a single endocrinologist, but it can also avoid the influence of differences in education methods and skills. Finally, the study period was relatively short. This could be one of the reasons for the results showing improved TIR and CV but not HbA1c. Therefore, further studies with a larger scale and longer duration are needed.

In conclusion, this RCT demonstrated the importance of ongoing, personalized education for the effective use of FGM in adults with type 1 diabetes. The remote intervention based on CGM data can be an effective educational model.

## Data availability statement

The datasets analyzed for this study are not publicly available but de-identified data may be made available upon request, subject to Institutional Review Board approval and a formal data use agreement. Contact the corresponding author for more information and access to these datasets.

## Ethics statement

The studies involving human participants were reviewed and approved by the Institutional Review Board of CHA Bundang Medical Center. The patients/participants provided their written informed consent to participate in this study.

## Author contributions

S-KK and YSS contributed to the conception and design of the trial. JL, MHL, K-SK, S-KK, Y-WC and YSS acquired patients and analyzed and interpreted the data. JL and YSS wrote the article and edited the manuscript. JL, MHL, JP, K-SK, S-KK, Y-WC, HWH and YSS approved the final manuscript. All authors contributed to the article and approved the submitted version.

## Funding

This research was supported by a grant from the Korea Health Technology R&D Project through the Korea Health Industry Development Institute (KHIDI), funded by the Ministry of Health & Welfare, Republic of Korea (No. HC20C0118), and a grant from the Information and Communications Promotion Fund through the National IT Industry Promotion Agency (NIPA), funded by the Ministry of Science and ICT (MSIT), Republic of Korea.

## Conflict of interest

The authors declare that the research was conducted in the absence of any commercial or financial relationships that could be construed as a potential conflict of interest.

## Publisher’s note

All claims expressed in this article are solely those of the authors and do not necessarily represent those of their affiliated organizations, or those of the publisher, the editors and the reviewers. Any product that may be evaluated in this article, or claim that may be made by its manufacturer, is not guaranteed or endorsed by the publisher.
