# Supplementary material for: FGM-based remote intervention for adults with type 1 diabetes: The FRIEND randomized clinical trial
Source: Front Endocrinol (Lausanne). 2022 Nov 25;13:1054697. doi: 10.3389/fendo.2022.1054697 (PMC9732659; doi:10.3389/fendo.2022.1054697)
Supplement: Supplementary file 1 [file DataSheet_1.docx]

Supplementary Material

# Supplementary Figures


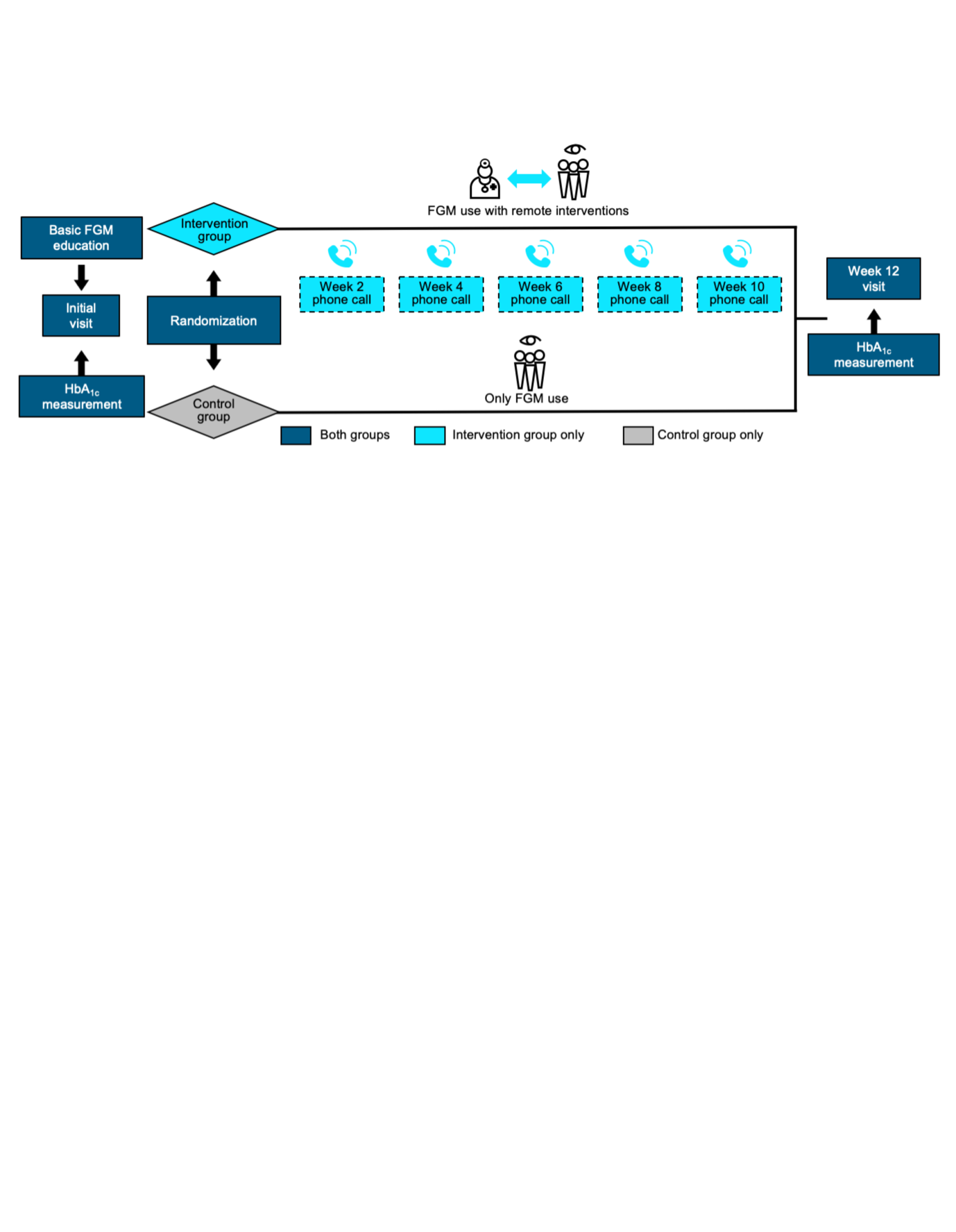


**Supplementary Figure 1. Schematic protocol of the FRIEND trial.**


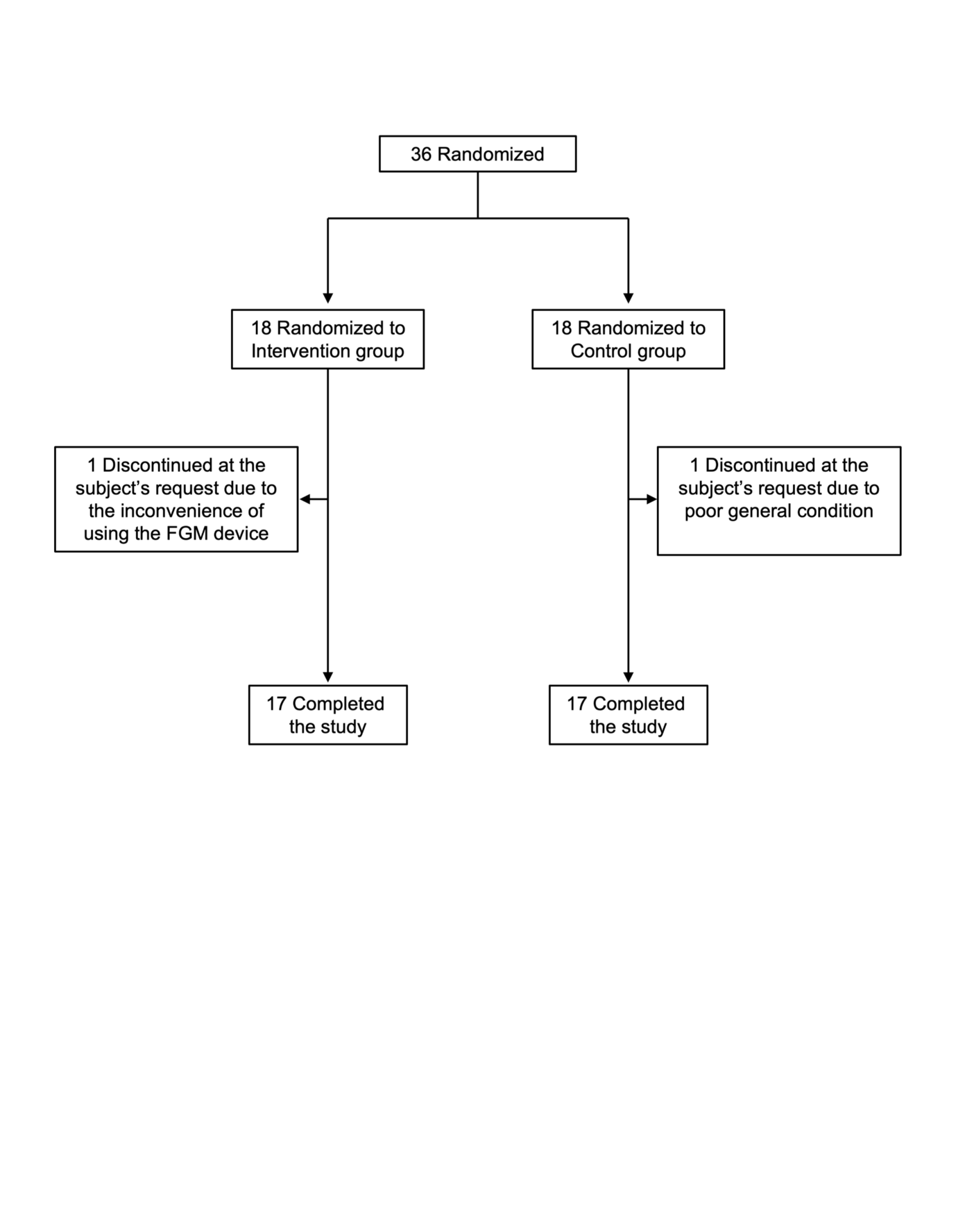


**Supplementary Figure 2. Flowchart of study participants.**

**
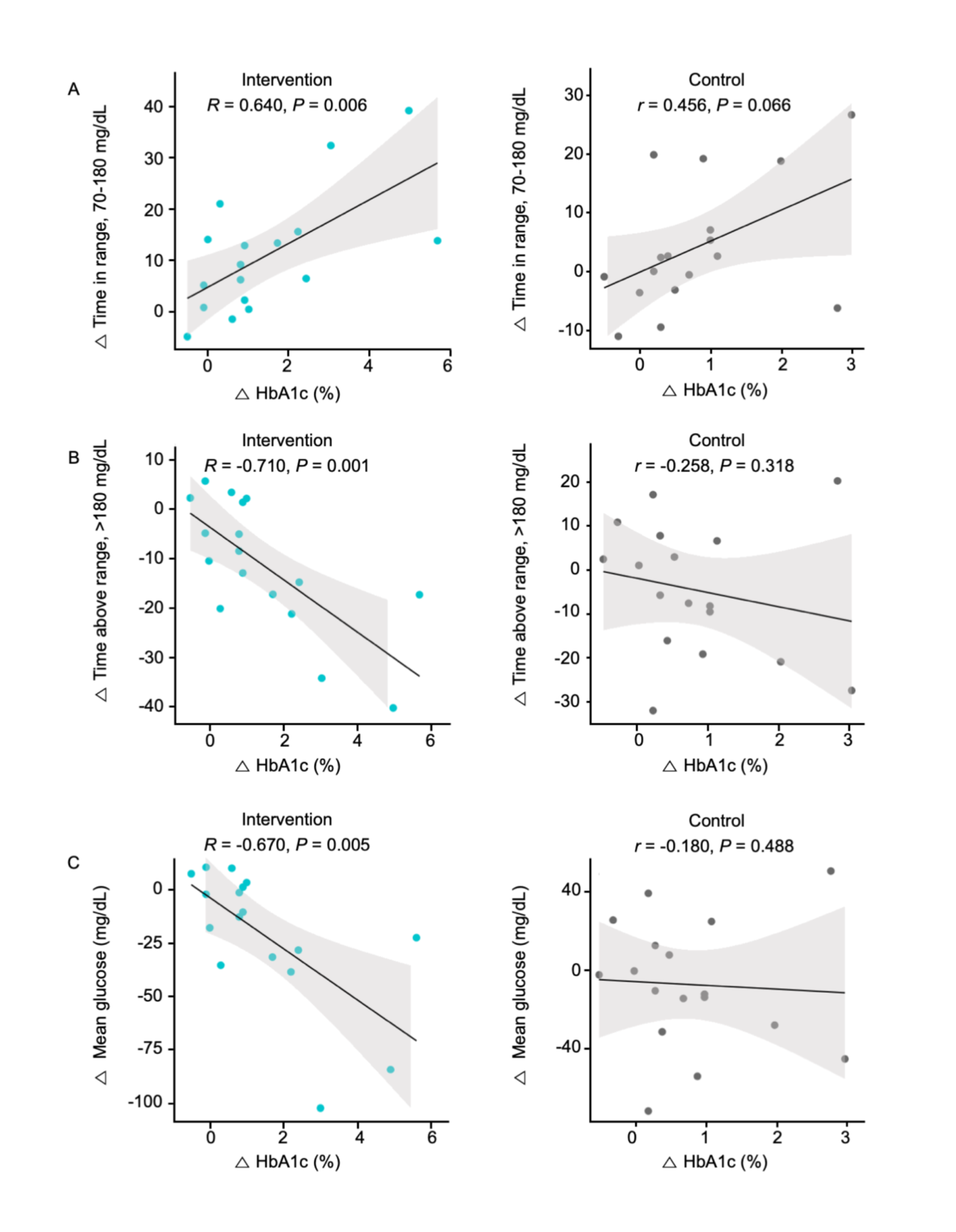
**

**Supplementary Figure 3. Correlation of changes in HbA1c with changes in continuous glucose monitoring metrics from baseline.**

Scatter plots depicting the relationship of the absolute values of mean HbA1c changes with the mean changes in time with glucose in ranges of 70–180 (A) and >180 mg/dL (B), and mean glucose (C). Pearson’s (*R*) or Spearman’s (*r*) correlation coefficients are presented with *P* values.

# Supplementary Tables

**Supplementary Table 1. Changes in continuous glucose monitoring metrics during daytime and nighttime**

|  | Baseline | Week 12 | Change from baseline (95% CI) | *P* | Adjusted difference between groups (95% CI) | *P* |
| --- | --- | --- | --- | --- | --- | --- |
| **Daytime (6:00 AM–11:59 PM)** |  |  |  |  |  |  |
| Time with glucose 70–180 mg/dL, % |  |  |  |  |  |  |
| Intervention | 49.2 ± 16.7 | 59.1 ± 7.9 | 9.9 (−0.4 to 20.3) | 0.060 | 6.0 (−2.6 to 14.5) | 0.164 |
| Control | 49.1 ± 17.1 | 53.2 ± 15.1 | 4.1 (−6.5 to 14.6) | 0.429 |  |  |
| Time with glucose <54 mg/dL, % |  |  |  |  |  |  |
| Intervention | 0.0 (0.0–0.7) | 0.0 (0.0–0.4) | 0.1 (−0.7 to 1.4) | 0.754 | −3.6 (−10.4 to 3.2) | 0.287 |
| Control | 0.1 (0.0–2.1) | 0.5 (0.0–1.4) | −0.7 (−5.2 to 1.3) | 0.727 |  |  |
| Time with glucose <70 mg/dL, % |  |  |  |  |  |  |
| Intervention | 2.3 (1.3–5.0) | 4.0 (1.4–6.1) | 1.3 (−0.7 to 4.0) | 0.159 | −1.8 (−9.0 to 5.4) | 0.610 |
| Control | 3.1 (1.8–10.4) | 4.6 (2.8–8.2) | 1.0 (−4.8 to 5.8) | 0.712 |  |  |
| Time with glucose >180 mg/dL, % |  |  |  |  |  |  |
| Intervention | 40.2 (37.2–46.4) | 36.4 (32.6–39.8) | −6.2 (−23.5 to −1.0) | **0.015** | −2.0 (−9.2 to 5.2) | 0.568 |
| Control | 39.7 (33.6–62.4) | 38.7 (30.6–41.2) | −7.6 (−17.5 to 4.7) | 0.190 |  |  |
| Time with glucose >250 mg/dL, % |  |  |  |  |  |  |
| Intervention | 19.8 ± 16.8 | 10.8 ± 5.0 | −9.1 (−18.4 to 0.3) | 0.056 | −7.8 (−16.2 to 0.6) | 0.068 |
| Control | 19.9 ± 16.2 | 18.6 ± 16.0 | −1.4 (−13.3 to 10.6) | 0.810 |  |  |
| Mean glucose, mg/dL |  |  |  |  |  |  |
| Intervention | 184.8 ± 41.0 | 162.3 ± 22.2 | –22.6 (–39.0 to –6.1) | **0.010** | −13.3 (−32.2 to 5.5) | 0.158 |
| Control | 181.4 ± 45.1 | 173.9 ± 43.1 | –7.5 (–25.9 to 10.9) | 0.399 |  |  |
| Coefficient of variation, % |  |  |  |  |  |  |
| Intervention | 39.5 ± 5.9 | 38.7 ± 7.1 | −0.8 (−3.1 to 1.6) | 0.500 | −5.6 (−10.9 to −0.3) | **0.039** |
| Control | 42.2 ± 10.3 | 42.6 ± 6.6 | 0.4 (−3.2 to 4.0) | 0.831 |  |  |
| **Nighttime (12:00 AM–5:59 AM)** |  |  |  |  |  |  |
| Time with glucose 70–180 mg/dL, % |  |  |  |  |  |  |
| Intervention | 51.6 ± 16.9 | 65.6 ± 17.0 | 14.0 (4.2 to 23.8) | **0.008** | 9.4 (−2.3 to 21.0) | 0.110 |
| Control | 52.1 ± 18.1 | 56.3 ± 16.6 | 4.3 (−7.6 to 16.2) | 0.459 |  |  |
| Time with glucose <54 mg/dL, % |  |  |  |  |  |  |
| Intervention | 0.3 (0.0–1.2) | 0.0 (0.0–1.5) | −0.2 (−8.1 to 5.9) | 0.944 | 0.7 (−5.6 to 7.1) | 0.814 |
| Control | 0.3 (0.0–2.1) | 0.0 (0.0–0.9) | −0.3 (−3.5 to 0.5) | 0.443 |  |  |
| Time with glucose <70 mg/dL, % |  |  |  |  |  |  |
| Intervention | 9.6 (1.6–22.1) | 4.2 (2.5–12.0) | −5.0 (−11.3 to 3.8) | 0.378 | −1.0 (−8.1 to 6.2) | 0.780 |
| Control | 4.9 (1.1–10.9) | 7.7 (1.7–11.6) | 1.5 (−5.2 to 7.7) | 0.678 |  |  |
| Time with glucose >180 mg/dL, % |  |  |  |  |  |  |
| Intervention | 36.9 ± 26.0 | 26.9 ± 13.0 | −10.0 (−25.4 to 5.4) | 0.187 | −8.6 (−20.3 to 3.1) | 0.145 |
| Control | 39.9 ± 18.0 | 35.4 ± 19.3 | −4.5 (−18.0 to 9.0) | 0.489 |  |  |
| Time with glucose >250 mg/dL, % |  |  |  |  |  |  |
| Intervention | 9.0 (7.1–14.8) | 3.0 (1.6–9.6) | −5.7 (−11.0 to −1.0) | **0.035** | −5.1 (−12.0 to 1.9) | 0.146 |
| Control | 13.8 (6.2–19.1) | 8.4 (3.7–15.8) | −3.8 (−12.0 to 7.0) | 0.611 |  |  |
| Mean glucose, mg/dL |  |  |  |  |  |  |
| Intervention | 164.1 ± 54.0 | 150.9 ± 31.6 | –13.2 (–34.3 to 7.9) | 0.204 | −6.7 (−25.3 to 11.9) | 0.466 |
| Control | 169.4 ± 42.0 | 160.2 ± 38.4 | –9.2 (–24.7 to 6.3) | 0.226 |  |  |
| Coefficient of variation, % |  |  |  |  |  |  |
| Intervention | 41.4 ± 8.8 | 36.3 ± 12.2 | −5.1 (−11.1 to 1.0) | 0.094 | −7.0 (−12.9 to −1.1) | **0.022** |
| Control | 40.3 ± 6.9 | 41.3 ± 6.9 | 1.0 (−1.5 to 3.6) | 0.395 |  |  |

Values are presented as the mean ± standard deviation or median (interquartile range). The change at week 12 from baseline in each group was evaluated using paired *t*-test or Wilcoxon signed-rank test for parametric or non-parametric data, and was presented as the mean or median, respectively. The baseline corrected difference between groups was evaluated using analysis of covariance (ANCOVA) or rank transform ANCOVA, depending on whether the ANCOVA assumptions were met, and was presented as the mean of values or mean residual of rank-transformed values, respectively. Significant *P* values in bold.

**Supplementary Table 2. Changes in continuous glucose monitoring metrics at 4-week intervals**

|  | Baseline | Week 4 | Week 8 | Week 12 | *P*-trend |
| --- | --- | --- | --- | --- | --- |
| Time with glucose 70–180 mg/dL, % |  |  |  |  |  |
| Intervention | 49.9 ± 15.9 | 53.7 ± 16.1 | 58.8 ± 14.0 | 61.1 ± 8.4 | **0.013** |
| Control | 49.8 ± 15.6 | 52.6 ± 20.2 | 53.7 ± 17.6 | 54.5 ± 14.2 | 0.418 |
| Time with glucose >180 mg/dL, % |  |  |  |  |  |
| Intervention | 44.3 ± 19.3 | 40.2 ± 18.2 | 35.1 ± 16.6 | 32.8 ± 12.1 | **0.034** |
| Control | 43.0 ± 18.5 | 39.7 ± 22.9 | 39.6 ± 19.2 | 38.0 ± 18.0 | 0.484 |
| Mean glucose, mg/dL |  |  |  |  |  |
| Intervention | 180.1 ± 43.3 | 172.4 ± 37.9 | 165.9 ± 36.6 | 158.8 ± 23.0 | 0.075 |
| Control | 178.6 ± 42.1 | 173.8 ± 48.5 | 172.9 ± 39.5 | 169.3 ± 40.5 | 0.539 |
| Coefficient of variation, % |  |  |  |  |  |
| Intervention | 40.8 ± 6.5 | 40.2 ± 7.1 | 39.9 ± 7.7 | 38.6 ± 7.5 | 0.359 |
| Control | 42.7 ± 9.3 | 41.1 ± 7.1 | 40.7 ± 9.0 | 43.2 ± 6.4 | 0.896 |

Values are presented as the mean ± standard deviation. One-way analysis of variance and test for linearity was performed to test linear trends. Significant *P* values in bold.
